# The Use of Nipple Shields: A Review

**DOI:** 10.3389/fpubh.2015.00236

**Published:** 2015-10-16

**Authors:** Selina Chow, Ronald Chow, Marko Popovic, Henry Lam, Joav Merrick, Søren Ventegodt, Milica Milakovic, Michael Lam, Mila Popovic, Edward Chow, Jelena Popovic

**Affiliations:** ^1^Toronto East General Hospital, Toronto, ON, Canada; ^2^Sunnybrook Health Sciences Centre, Toronto, ON, Canada; ^3^Health Services, Division for Intellectual and Developmental Disabilities, Ministry of Social Affairs, National Institute of Child Health and Human Development, Jerusalem, Israel; ^4^Quality of Life Research Center, Copenhagen, Denmark

**Keywords:** nipple shield, breastfeeding, lactation

## Abstract

**Introduction:**

A nipple shield is a breastfeeding aid with a nipple-shaped shield that is positioned over the nipple and areola prior to nursing. Nipple shields are usually recommended to mothers with flat nipples or in cases in which there is a failure of the baby to effectively latch onto the breast within the first 2 days postpartum. The use of nipple shields is a controversial topic in the field of lactation. Its use has been an issue in the clinical literature since some older studies discovered reduced breast milk transfer when using nipple shields, while more recent studies reported successful breastfeeding outcomes. The purpose of this review was to examine the evidence and outcomes associated with nipple shield use.

**Methods:**

A literature search was conducted in Ovid MEDLINE, OLDMEDLINE, EMBASE Classic, EMBASE, Cochrane Central Register of Controlled Trials, and CINAHL. The primary endpoint was any breastfeeding outcome following nipple shield use. Secondary endpoints included the reasons for nipple shield use and the average/median length of use. For the analysis, we examined the effect of nipple shield use on physiological responses, premature infants, mothers’ experiences, and health professionals’ experiences.

**Results:**

The literature search yielded 261 articles, 14 of which were included in this review. Of these 14 articles, three reported on physiological responses, two reported on premature infants, eight reported on mothers’ experiences, and one reported on health professionals’ experiences.

**Conclusion:**

Through examining the use of nipple shields, further insight is provided on the advantages and disadvantages of this practice, thus allowing clinicians and researchers to address improvements on areas that will benefit mothers and infants the most.

## Introduction

The immunologic and anti-infective properties of breast milk are advantageous to babies, particularly high-risk premature infants (1). Moreover, breastfeeding establishes important emotional and bonding experiences for the mother–infant dyad (2).

Since breastfeeding confers benefits to both mothers and infants, it is necessary to promote breastfeeding and mitigate barriers that may prevent its success and/or lead to early breastfeeding termination (3). For example, the reluctant or non-nursing infant is an overwhelming challenge to a new mother (4). Many women in this situation wean their breastfeeding efforts due to the absence of timely help or the lack of resources/support (4). When maternal and/or infant-related factors challenge breastfeeding, nipple shields may preserve and facilitate breastfeeding (3).

A nipple shield is a breastfeeding aid with a nipple-shaped shield that is positioned over the nipple and areola prior to nursing (3). Nipple shields are usually recommended to mothers for flat nipples or in cases in which there is a failure of the baby to effectively latch onto the breast within the first 2 days postpartum. They are also used for sore nipples, prematurity, oversupply, transitioning infants from the bottle to the breast, and other indications (5).

The physical design of the shield has drastically changed over time, dating back to the sixteenth century (6). Nipple shields have progressed from being made of lead, wax, silver, wood, pewter, and animal skins, to rubber, thin latex, and today’s silicone models (5–7).

In order to use a nipple shield effectively, it should correctly fit the mother’s breast, and the infant should be latched onto the entire areola, not just the shield’s tip. The shield needs to be positioned over the center of the nipple. A series of clockwise rotations should then guide the nipple into the shield tunnel and stretch the shield’s base around the areola. Each stretch of the shield draws more nipple tissue into the shield. The edges of the shield circumference can be secured over the areola with a few drops of water. If the infant is latched onto the shield properly, each suck will show visible movements in the area of the breast distal to the shield. In contrast, little or no breast movement is visible with sucking if the infant is only on the tip of the nipple shield (8).

The use of nipple shields is a controversial topic in lactation. Its use has been an issue in the clinical literature since some older studies discovered reduced breast milk transfer when using nipple shields (9–12). Nonetheless, more recent studies have reported successful breastfeeding outcomes following the use of nipple shields (4, 7, 13–18).

Nipple shields are not only debated among healthcare professionals but also among mothers. The shields may act as a solution to a problem, thus reducing the stress from breastfeeding difficulties, or it may increase stress when women aim to breastfeed without accessories (18). To provide a foundation of evidence for the use of nipple shields, this review was undertaken to evaluate the evidence and outcomes associated with nipple shield use.

## Methods

**Figure 1:**
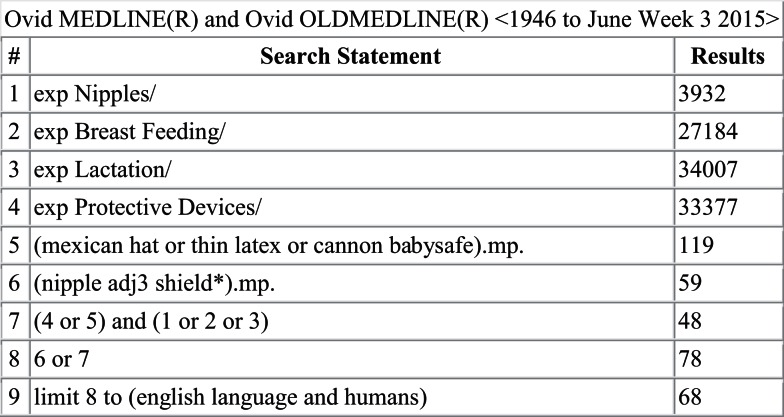
**Search strategy for Ovid MEDLINE and OLDMEDLINE**.

**Figure 2:**
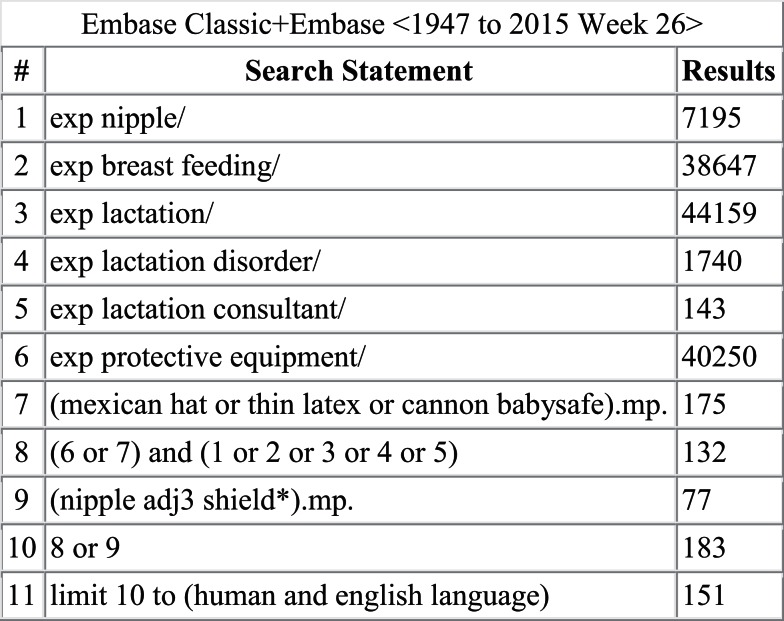
**Search strategy for EMBASE Classic and EMBASE**.

**Figure 3:**
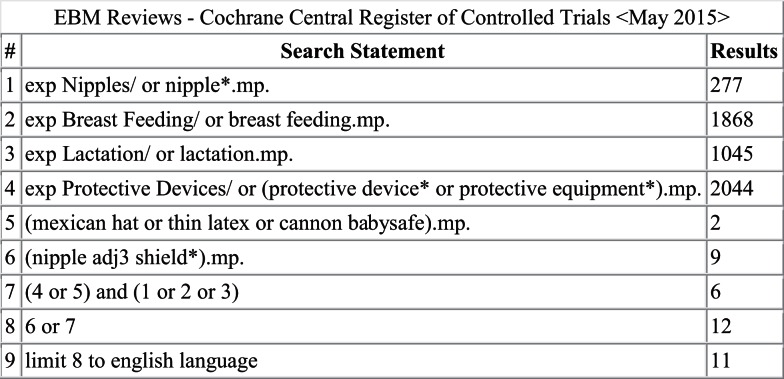
**Search strategy for Cochrane Central Register of Controlled Trials**.

**Figure 4:**
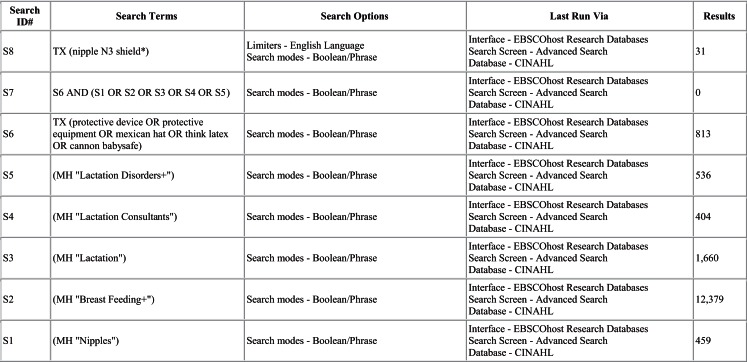
**Search strategy for CINAHL**.

A literature search was conducted in Ovid MEDLINE and OLDMEDLINE (1946 to June Week 3 2015), EMBASE Classic and EMBASE (1947 to 2015 Week 26), Cochrane Central Register of Controlled Trials (up until May 2015), and CINAHL (up until July 1, 2015). A full list of search terms is provided in Figures 1–4. Titles and abstracts were screened to identify if studies were relevant for full-text screening, after which full texts were included if they met the pre-specified inclusion criteria.

### Selection Criteria

Articles were selected for full-text screening if the title or abstract mentioned nipple shield(s). Only English language studies were included. Duplicates of articles found in each database, as well as non-original research, small (i.e., <5 patients) sized studies, and research on nipple shield use for anything other than breastfeeding (e.g., delivery system for antiviral agents preventing HIV transmission, reconstructive surgery, cancer treatment) were excluded.

### Data Extraction and Endpoints

The primary endpoint was any breastfeeding outcome following nipple shield use. Secondary endpoints included the reasons for nipple shield use and the average/median length of use. For the analysis, we examined the effect of nipple shield use on physiological responses, premature infants, mothers’ experiences, and health professionals’ experiences.

## Results

The literature search yielded 261 articles, of which 68 were from MEDLINE, 151 from EMBASE, 11 from Cochrane Central, and 31 from CINAHL. Of those, 31 articles were identified for full-text review as specified by the inclusion criteria; 17 of the 31 articles were rejected after full-text review. Some of the reasons for exclusion were the lack/absence of relevant information regarding breastfeeding outcomes with nipple shield use as well as editorials and case reports. Of the 14 remaining articles (2–4, 7, 9, 10, 12–14, 16–20), three reported on physiological responses (9, 10, 12), two reported on premature infants (2, 16), eight reported on mothers’ experiences (3, 4, 7, 13, 14, 17–19), and one reported on health professionals’ experiences (20).

### Physiological Responses

Three studies reported on the physiological responses during breastfeeding with a nipple shield (9, 10, 12).

Amatayakul et al. (10) randomly assigned 50 Northern Thai women to one of three groups: group 1 (16/50) breastfed without a thin latex nipple shield, group 2 (16/50) breastfed with a thin latex nipple shield, and group 3 (18/50) wore a thin latex nipple shield but did not breastfeed. At 1 week postpartum, prolactin and cortisol levels, infant suckling time, and milk transfer were measured with and without a nipple shield. Based on blood samples collected before, during, and after the feeding, no significant differences in either hormone levels were found between groups 1 and 2 (prolactin – *p* = 0.83; cortisol – *p* > 0.1). Use of the nipple shields when breastfeeding had significantly reduced milk transfer, from a median of 47 g in group 1 to a median of 27 g in group 2, which was likely due to the inhibition of oxytocin release in group 2 mothers (10) (Table 1).

**Table 1:** **Physiological responses with nipple shield use**.

Author	Study population	Methods	Outcomes
Amatayakul et al. (10)	• 50 Northern Thai women • Were patients at the delivery wards of the University Hospital, Chiang Mai, or from the Mother and Child Health Centre • Inclusion criteria – Were breastfeeding satisfactorily – Had breastfed at least 1 previous child – Normal labor – No complications after delivery – Baby was healthy and free from complications – Baby weighed 3000–3500 g	• Randomly assigned to 1 of 3 groups – Group 1 (16/50) breastfed without a nipple shield – Group 2 (16/50) breastfed with the nipple shield – Group 3 (18/50) wore a nipple shield but did not breastfeed • If babies were nursed in the study, they were only fed on the left breast • Measured prolactin and cortisol levels, infant suckling time, and milk transfer by test weighing with and without a thin latex nipple shield at 1 week postpartum	• Infant suckling time not significantly different between groups 1 and 2 – Median for group 1: 11 min – Median for group 2: 12 min – Range of 8–16 min for both • Highly significant change of prolactin levels over time • Cortisol levels declined slowly over time, yielding a highly significant change • Based on blood samples collected before, during, and after the feeding, no significant differences in prolactin and cortisol levels between groups 1 and 2 • Thin latex nipple shield had no impact in hormone release during breastfeeding • No evidence for release of prolactin/cortisol when the shield was in place without suckling • No significant association between time spent suckling and prolactin levels at 5, 10, 20, or 30 min – Association significant at 40, 90, and 120 min, and borderline at 60 min • Significantly reduced milk transfer with nipple shield use – Median milk transfer to infants in group 1 was 47 g, whereas group 2 was 27 g – Due to the likely inhibition of oxytocin release in the group 2 mothers
Auerbach (12)	• 25 women with well-established lactation courses and thriving infants • Women who were pumping their breasts during/in anticipation of employment-related absences	• Each study subject participated in 2 different pumping sessions – 1 session involved 3 separate pumping periods on the right breast, each separated by a 5-min resting period – The same was done on the left breast for the other session • Pumping regimens consisted of 3 consecutive 5-min periods per breast – Without nipple shield – With old design of shield – With new design of shield • Milk was pumped from the breast by a standard size, high-quality, intermittent electric pump	• Pumping without a shield resulted in mean volumes 6 times greater than when the old shield was used and 4 times greater than when the new shield was used • The new shield appeared to negatively affect milk volume slightly less than the old shield – 17 versus 12% of overall volume
Woolridge et al. (9)	• Inclusion criteria – Trouble-free lactation – Age of babies to be 5–8 days, inclusive Mexican Hat • 16 mother–infant dyads Thin latex • 18 mother-infant dyads	• Milk intake was assessed from the baby ’s weight gain – Measured by test weighing • Sucking patterns were determined by filming the mouth of the baby during the feed	• Mexican Hat reduced milk transfer by 58% – Mean volume of 19.5 g compared to a mean volume of 46.4 g without a nipple shield • Thin latex shield reduced milk transfer by a smaller amount (22%) – Mean volume of 29.9 g compared to a mean volume of 38.4 g without a nipple shield • Infant suckling patterns were significantly altered when a Mexican Hat was in place – Mexican Hat increased sucking rate (i.e., mean inter-suck interval was shorter) and the time spent resting (the length of the pauses were not increased; there were just more of them) • Little difference (e.g., sucking frequency or pauses) was observed when mothers used the thin latex nipple shield

*g, grams*.

Auerbach (12) also examined milk transfer with a nipple shield. Twenty-five mothers participated in two separate pumping sessions, one for each breast, where different designs of nipple shields were tested. The “old” shield was the Cannon Babysafe (Glemsford, UK) with four small holes, and the “new” shield was the modified design with one hole. Pumping without a shield yielded larger amounts of milk, with mean volumes six times greater than when the old shield was used and more than four times greater than when the new shield was in place. Evidently, the new shield seemed to reduce the milk volume slightly less than the old shield (17% versus 12% of overall volume), although this difference was not statistically significant (12) (Table 1).

Woolridge et al. (9) compared the Mexican Hat nipple shield and the thin latex nipple shield with 16 and 18 mother-infant dyads, respectively, at 5–8 days postpartum. It was found that both nipple shields reduced milk transfer: the Mexican Hat decreased milk supply by 58%, with a mean volume of 19.5 g compared to a mean volume of 46.4 g without a shield, whereas the thin latex shield diminished milk intake by 22% from a mean volume of 38.4 g without a shield to 29.9 g. Recorded videos of the babies’ mouths during the feeding process revealed that infant suckling patterns were significantly altered when a Mexican Hat was in place. This nipple shield design increased sucking rate and the time spent resting. In contrast, minimal differences in sucking frequency and pauses were observed when using the thin latex nipple shield (9) (Table 1).

### Premature Infants

Two studies reported the breastfeeding outcomes with nipple shield use for premature infants (2, 16).

Clum and Primomo (2) performed chart reviews for 15 premature infants who were neonatal intensive care unit (NICU) patients and whose mothers intended to breastfeed. In order to investigate the effect of nipple shield use on milk transfer, the infants’ prescribed amount of feeding was compared to their actual intake, which was measured by test weights. It was identified that health professionals usually recommended nipple shields if the neonate had difficulty latching for an average of 5 days. The average gestational age at first nipple shield use was 34.9 weeks, ranging from 33 to 39 weeks. Using a nipple shield, nine infants (60%) consumed 50% or more of the prescribed feeding amount, and six infants consumed between 13 and 28% of the prescribed feeding amount. Therefore, the majority of patients obtained at least half of the prescribed feeding amount during their first nipple shield use, which is an acceptable amount for preterm babies transitioning from gavage to breast/bottle-feeding (2) (see Table 2).

**Table 2:** **Effects of nipple shield usage on premature infants**.

Author	Study population	Methods	Outcomes
Clum and Primomo (2)	• 15 premature infants • Patients at a NICU in South Puget Sound • Mothers intended to breastfeed	• Charts were reviewed to identify – Maternal parity – Gestational age – Birth weight – Age of infant at first feed – Age at introduction of nipple shield – Infant age at discharge • Prescribed amount of feeding was compared to the actual intake by test weights • Mothers maintained their milk supply with a high-quality, intermittent electric pump with a known pressure – Were advised to pump both breasts simultaneously for 10 min, 8 times every 24 h	• Reasons for nipple shield use – Had difficulty latching without the shield for an average of 5 days • Infants’ average gestational age at first nipple shield use: 34.9 weeks (range of 33–39 weeks) • 9 infants (60%) consumed ≥50% of the prescribed feeding amount using a nipple shield • 6 infants consumed 13–28% of the prescribed feeding amount using a nipple shield
Meier et al. (16)	• 34 premature infants • Were hospitalized in 1 of 2 hospitals during a 12-month period in 1997–1998 • Mothers had used nipple shields to facilitate milk intake during and/or after each infant’s stay in a NICU	• Volume of milk transfer, measured by infant test-weights, was compared for 2 consecutive breastfeeding with and without the nipple shield • Total duration of nipple shield use and breastfeeding were calculated • Reasons for nipple shield use were recorded	• Reasons for nipple shield use – Poor latch [21/34 (61.8%)] • Slipping off the nipple during pauses in sucking, large/flat nipples difficult for the infant to achieve and/or sustain an effective breastfeeding position – Correct infants’ falling asleep within minutes of being positioned at the breast [10/34 (29.4%)] – Maternal nipple discomfort [3/34 (8.8%)] • All infants consumed more milk with the shield – Mean transfer of milk without a shield was 3.9 mL, compared to 18.4 mL with the shield – Mean of 14.4 mL difference • Mean duration of nipple shield use: 33 days (range of 2–171 days) – Used for a mean of 24.3% (range of 0.6%–100%) of total breastfeeding experience

*mL, milliliter; NICU, neonatal intensive care unit*.

Meier et al. (16) performed a retrospective analysis of data for 34 premature infants who were NICU patients and whose mothers had used nipple shields to facilitate milk intake during and/or after each infants’ stay in the NICU. This study examined the effect of nipple shields on milk transfer and total duration of breastfeeding. The volume of milk transfer, which was measured by infant test weights, was compared for two consecutive breastfeeding (one with and one without the use of a nipple shield). It was found that poor infant latch [21/34 (61.8%)], infants falling asleep soon after being positioned at the breast [10/34 (29.4%)], and maternal nipple discomfort [3/34 (8.8%)] were all reasons for nipple shield use. When using the shield, all infants consumed more milk than without nipple shields. The mean transfer of milk without a shield was 3.9 mL, compared to a mean of 18.4 mL with the shield, resulting in a 14.4 mL difference. These infants used the nipple shield for a mean duration of 33 days, which was a mean of 24.3% of the total breastfeeding experience (16) (Table 2).

### Mothers’ Experiences

Eight studies focused on the mothers’ experiences with nipple shield use (3, 4, 7, 13, 14, 17–19). Of these studies, four were prospective (3, 13, 18, 19) and four were retrospective (4, 7, 14, 17).

Chertok et al. (18) conducted a prospective two-part pilot study. Part 1 consisted of 32 breastfeeding mother–infant dyads that had received support from lactation consultants and had used or were still using nipple shields. A structured telephone survey was used to examine maternal satisfaction with nipple shield use. The reasons for nipple shield use were for infant reasons [16/32 (50%)], maternal reasons [12/32 (37.5%)], and both maternal and infant reasons [4/32 (12.5%)]. Overall, mothers were satisfied with nipple shields and attributed its use with preventing early weaning (18) (Table 3). This study’s second part used a within-subject design to evaluate maternal prolactin and cortisol levels and infant test weights during two breastfeeding sessions, one with and one without the nipple shield. The study population included five maternal–infant dyads that had completed Part 1 and were in the process of weaning from nipple shield use. Based on blood samples collected immediately before, and 10 and 20 min after breastfeeding started, maternal hormone levels were not significantly different for breastfeeding sessions with and without the nipple shield (prolactin – *p* = 0.88; cortisol – *p* = 0.74). Similarly, there were no significant differences in infant breast milk intake for breastfeeding sessions with and without nipple shields (*p* = 0.72). Therefore, nipple shields were an effective intervention strategy that did not affect milk transfer or hormone levels and could prevent early breastfeeding termination (18) (Table 3).

**Table 3:** **Mothers’ experiences with nipple shield usage**.

Author	Study population	Methods	Outcomes
Bodley and Powers (7)	• 10 mothers	• Chart reviews	• Reasons for nipple shield use – Inability to grasp the areola (7/10) • Due to suck difficulties or poor protractility of breast tissue – Sore nipples (1/10) – Both (2/10) • Women used the shield long-term for 2 weeks to 3.5 months – All study subjects tried to eliminate the shield within a few days of starting its use – Eventually all babies quit nursing through the shield • 2 mothers used the shield on 1 nipple only • None of the mothers supplemented with artificial baby milk or pumped breast milk while using the nipple shield • From the first weight check at 3–8 days to the 3-week check, all babies had an appropriate weight gain • At the 2-month check, weight gain was appropriate, if not abundant, for all 10 babies • At the 4-month check, weight gain was appropriate for all infants – 9 babies were feeding directly from the breast at this time, and 1 was bottle-feeding • 9 mothers were extremely positive about the use of the shield to help in their situations – The 10th mother felt the shield was inconvenient, but it was a tool which helped her achieve her goal of breastfeeding
Brigham (14)	• 51 clients of the Breastfeeding Center at Evergreen Hospital • Were given a nipple shield in 1994 • Infant population included healthy, term infants, premature infants, and infants with Down syndrome	• Study subjects were interviewed for an average of about 10 min by telephone • The following information was documented – Reason for nipple shield use – Age of baby at first use – Length of use – Duration of breastfeeding (total duration and duration after shield discontinued) – Helpfulness of the shield	• Reasons for nipple shield use – Difficulty with latch [37/51 (73%)] • Flat nipples [11/37 (30%)] • Inverted nipples [6/37 (16%)] • Engorgement [5/37 (14%)] • Nipple confusion [3/37 (8%)] • Premature infant [1/37 (3%)] • Infant with Down syndrome [1/37 (3%)] • Weak infant suck [1/37 (3%)] • Infant with retracted tongue [1/37 (3%)] – Sore nipples [5/51 (10%)] – Both [9/51 (18%)] • Average age of infants when the nipple shield was initiated: 6.1 days (range of 1–42 days) • Average length of shield use: 26.7 days (range of 2 days–4.5 months) • 86% (44/51) of respondents reported that the nipple shield helped them continue to breastfeed – 7 women did not find the shield helpful and discontinued using it – Although breastfeeding duration was short for some, some mothers reported satisfaction that at least some breastfeeding was possible, which they felt would not have occurred without the shield • No one identified insufficient milk supply or poor infant growth patterns with nipple shield use
Chertok et al. (18)	Part 1 • 32 breastfeeding mother–infant dyads • Received support from lactation consultants at Evergreen Hospital or University of Washington Medical Center • Inclusion criteria – Healthy postpartum women – Knowledge of English – Delivered by vaginal/cesarean delivery – Had a healthy, full-term (37–42 weeks) infant singleton – Practiced exclusive breastfeeding (no supplementation) or nearly exclusive breastfeeding (minimal fluid supplementation) – Had used/were still using nipple shields Part 2 • 5 mother–infant dyads • Had completed Part 1 • Were in the process of weaning from the nipple shield	Part 1 • A structured, 15–20 min, maternal nipple shield satisfaction telephone survey • To examine – Maternal and infant demographics – Previous breastfeeding experience – Current breastfeeding and pumping experience – Nipple shield use – Infant feeding – Supplementation and use of pacifier – Infant weight gain history Part 2 • A prospective within-subject design • Used maternal and infant physiological outcomes to examine maternal prolactin and cortisol levels and infant test weights during 2 breastfeeding sessions with and without the nipple shield • Hormone levels were analyzed by collecting 3 blood samples – Immediately before – 10 min – 20 min after breastfeeding commenced	Part 1 • Reasons for nipple shield use – Infant reasons [16/32 (50%)] • Poor suck, poor latch, tongue displacement, etc. – Maternal reasons [12/32 (37.5%)] • Nipple pain, nipple trauma, breast engorgement, inverted/flat nipples, etc. – Both [4/32 (12.5%)] • Most women [26/32 (81.3%)] reported having no nipple pain with nipple shield use • Remaining women reported nipple soreness with use of the nipple shield • 62.5% (20/32) of women reported no complications with nipple shield use • 37.5% (12/32) of women reported that the nipple shield complicated breastfeeding – Types of complications • Nipple shield tended to fall off the breast (5/32) • Inconvenience (3/32) • Infant dependency on the shield (2/32) • Infant swallowed too much air (2/32) • Messiness (1/32) • Average length of shield use: 7.3 days (range of 3–13 days) • If they did not use the nipple shield – 6 would terminate breastfeeding – 6 would pump breast milk – 16 would continue trying to breastfeed – 4 expressed concerns over continued infant weight loss Part 2 • Maternal prolactin and cortisol levels for breastfeeding sessions with and without the nipple shield were not significantly different • No significant differences in the mean prolactin levels – Levels significantly increased over time for breastfeeding with and without nipple shields • No significant differences in the mean cortisol levels – No significant change over time – Levels with and without nipple shields followed similar trends over time • No significant differences in maternal hormonal levels and infant breast milk intake for breastfeeding sessions with and without nipple shields
Chertok (3)	• 54 mother–infant dyads • From 2 major cities in USA and Israel • Inclusion criteria – Healthy mothers – Term, healthy infants – Experience with breastfeeding – Experience using nipple shields during the postpartum period	• Study subjects were interviewed with 10 questions by telephone • To examine – Lactation practices – Nipple shield use – Infant weight gain over 2 months postpartum – Maternal satisfaction • Mothers were surveyed at birth and 2 weeks, 1 month, and 2 months postpartum	• Reasons for nipple shield use – Maternal reasons (61%) • Flat/inverted nipples, nipple pain, nipple trauma, engorgement, etc. – Infant reasons (39%) • Poor/weak latch/suck, etc. • 15% (8/54) of women had complications with nipple shield use – Types of complications • Shield falls off the areola [3/54 (37.5%)] • Nipple soreness [2/54 (25%)] • Inconvenience [2/54 (25%)] • Messiness [1/54 (12.5%)] • By 2 months postpartum, 65% (34/54) of women discontinued nipple shield use by the mean time of 2.96 (SD 2.1) weeks • Reasons for stopping nipple shield use – Improved breastfeeding, which ended the need for the shield [20/54 (56%)] – Cessation of lactation [6/54 (16.7%)] – Breastfeeding termination with continued pumping [6/54 (16.7%)] – Inconvenience [3/54 (8.3%)] – Recommendation of healthcare professional [1/54 (2.8%)] • Infant weight gain was similar for those using and not using nipple shields for 2 weeks • 89.8% of women had a positive experience with nipple shield use • 67.3% of women said that the nipple shield helped prevent breastfeeding termination
Nicholson (13)	• Study population was divided into 3 groups “NS” • 186 mothers • Seen by the hospital lactation consultant before discharge • Were using nipple shields “No NS” • 636 mothers • Seen by the hospital lactation consultant before discharge • Were not using nipple shields “PN” • 349 breastfeeding postnatal mothers • Not seen by the lactation consultant	• Collected data from all 3 groups before hospital discharge and 3 months postpartum, during 1988 and 1989 • A 3-month interview was carried out by telephone or a questionnaire was sent by mail • Following information was recorded • Feeding method at 3 months • Problems (mastitis and nipple trauma) experienced between hospital discharge and 3 months	• Breastfeeding rates on discharge – “NS”: 95% (176/186) – “No NS”: 83% (530/636) – “PN”: 91% (318/349) • Breastfeeding continuation rates at 3 months postpartum – “NS”: 55% (92/166) – 51% exclusively (84/166) – “No NS”: 63% (298/473) – 54% exclusively (256/473) – “PN”: 67% (190/282) – 57% exclusively (161/282) • “NS”: 13 out of the 92 women breastfeeding at 3 months were still using nipple shields – All of these women were breastfeeding exclusively – 7 women used the nipple shield for their entire lactation • Breastfeeding problems at 3 months postpartum – “NS”: 9% (15/166) – nipple trauma; 12% (20/166) – mastitis – “No NS”: 6% (27/473) – nipple trauma; 8% (40/473) – mastitis – “PN”: 7% (19/282) – nipple trauma; 7% (19/282) – mastitis
Pincombe et al. (19)	• 317 women • Had given birth to their first baby (at term) in a large teaching maternity hospital in Adelaide, South Australia, between March and November 2003 • Inclusion criteria – Women ≥18 years of age – Primiparous – ≥37 weeks gestation – Intending to breastfeed – Able to understand and communicate in both written and spoken English	• BFHI Step 9 (giving no artificial teats/pacifiers to breastfeeding babies) was investigated during telephone interviews • 3 separate questions relating to the use of nipple shields, pacifiers/dummies, and bottle-feeds at 1 week, 6 weeks, 3 months, and 6 months postpartum • Participants were asked if they were still breastfeeding, and if they were breastfeeding fully (breast milk only) or partially (formula and/or solids and breast milk) • If baby had been weaned, the mother was asked the age of her baby (to the nearest week) when he/she was weaned	• 14.2% of mothers used a nipple shield while in the postnatal ward, while 85.8% did not • Higher rate of weaning was found among mothers who used artificial nipples, including nipple shields, compared to those who offered the breast exclusively • Breastfeeding duration was shorter for mothers who did not experience all of the BFHI practices (e.g., using no artificial nipples including a nipple shield, feeding >1 h of birth, receiving feeding assistance, giving only breast milk to the infant, rooming-in) compared to those women who experienced all of these practices • Unadjusted hazard ratio for weaning is 2.1 times greater for babies whose mothers used nipple shields compared with those who did not – 1.6 times greater for babies offered dummies/pacifiers while in the postnatal ward – 1.4 times greater for babies given a bottle feed • Increased hazard of weaning was found for mothers who were shown how to initiate breastfeeding by the midwife • Breastfeeding on demand while in hospital had a significantly increased risk of weaning
Powers, Tapia (17)	• 202 breastfeeding women • Inclusion criteria – Discontinued nipple shield use for a minimum of 1 week	• 10 min telephone survey assessing mothers’ perceptions regarding use of a silicone nipple shield and its impact on their breastfeeding experience • Data obtained were based on subjective recall of the women interviewed	• Reasons for nipple shield use – Short/flat nipples [125/202 (62%)] – Infant’s disorganized suck [88/202 (43%)] – Sore nipples [49/202 (23%)] – Engorgement [31/202 (15%)] – Prematurity [25/202 (12%)] – Short frenulum [4/202 (1%)] – Other reasons [3/202 (1%)] • Infant with a receding chin • Protecting burn scars on the mother’s areola • A mother who believed her infant’s difficulty with latch were because of her infant’s later diagnosed autism • 46% of women gave >1 reason for using a shield • Nipple shield use began the 1st–42nd day of the infant’s life – 60% (122/202) began nipple shield use on the first or second day after delivery – 97% (197/202) began within the first 2 weeks postpartum • Median duration of nipple shield use: 2 weeks • One third (67/202) used the nipple shield the entire time they breastfed (range of 1 day–15 months) • 92 women were given information regarding the shields for flat, inverted, or sore nipples – 67% (62/92) of these women chose to wear the shields – Only 51% of these 62 women believed that wearing them helped them to succeed at breastfeeding – Those who did not believe they were helpful commented that the nipple shields were uncomfortable • This was especially true after milk onset occurred, usually the third or fourth day postpartum, and the shields exacerbated leaking • 11% (22/202) of the women reported that the infant could have nursed without the nipple shield at any time, but they chose to use the shield for nipple pain or general comfort • 5% of women used the nipple shield on only one nipple • 88% (178/202) of mothers felt that the nipple shield helped them succeed at breastfeeding
Wilson-Clay (4)	• 32 women seen during a 13-month period between December 1992 and January 1994 in a private lactation clinic in Austin, TX, USA • Received nipple shields	• Chart reviews	• 75% of mothers were already feeding with bottles (some exclusively) at consultation • Most common presenting problems – Breast refusal (69%) – Difficulty with latch (25%) – Sore nipples (6%) • 50% of mothers had flat/inverted nipples • 41% of mothers had breast engorgement upon presentation • Weaning of nipple shields – Initial crisis period (<6 weeks) [12/32 (38%)] – After 6 weeks [18/32 (56%)] – Fed human milk by bottle [2/32 (6%)]

*BFHI, baby friendly hospital initiative; SD, standard deviation*.

Chertok (3) conducted another telephone survey in 2009, which involved 54 maternal–infant dyads from the United States of America and Israel, who had experienced nursing with and without nipple shields during the postpartum period. Mothers were surveyed at birth and 2 weeks, 1 month, and 2 months postpartum in order to determine how nipple shield use affected infant weight gain. Reasons for nipple shield use were mostly maternally related (61%) but also sometimes infant related (39%). Infant weight gain was similar for those using and not using nipple shields at the 2-week survey (*p* = 0.30 and *p* = 0.16, respectively). In total, 89.8% of mothers had a positive experience with nipple shields and 67.3% credited the nipple shields for prevention of breastfeeding discontinuation (3) (Table 3).

Nicholson (13) conducted a prospective study in which the study population was divided into three groups: “NS” – 186 mothers who were seen by the hospital lactation consultant and were given nipple shields; “No NS” – 636 mothers who were seen by the hospital lactation consultant and were not given nipple shields; “PN” – 349 breastfeeding postnatal mothers who were not seen by the lactation consultant. Data were collected from all groups before hospital discharge and at 3 months postpartum. A 3-month interview was carried out by telephone or a questionnaire was sent by mail to investigate the feeding method at 3 months and problems experienced between hospital discharge and 3 months postpartum. Although “No NS” mothers had a much lower breastfeeding rate on discharge than the “NS” group (*p* < 0.001), this significant difference disappeared at 3 months postpartum. Since there were not any significant differences in breastfeeding rates at 3 months between the “NS” and “No NS” group, nipple shield use was not considered to have negative effects on lactation. Significantly fewer “NS” mothers were breastfeeding at 3 months (55%) than those in the “PN” group (67%) (*p* = 0.01). It was found that more than half of the women in each group continued breastfeeding at 3 months, and the majority were breastfeeding exclusively. There was a small proportion of mothers in all groups who experienced nipple trauma and mastitis at 3 months; the “NS” group had the highest rates of breastfeeding problems, followed by “No NS” and “PN.” Therefore, use of a nipple shield did not impede breastfeeding initiation (13) (Table 3).

In a final prospective study, Pincombe et al. (19) assessed the effects of Baby Friendly Hospital Initiative (BFHI) procedures on breastfeeding duration. Three hundred seventeen mothers who were intending to breastfeed and had given birth to their first at term baby in an Australian hospital were included in the study. BFHI Step 9, which is to restrain from giving artificial teats/pacifiers to breastfeeding babies, was analyzed through telephone interviews consisting of three separate questions. A total of 14.2% of participants used a nipple shield while in the postnatal ward. A higher rate of weaning was found among mothers who used artificial nipples (e.g., nipple shields) compared to mothers who offered the breast exclusively. Other factors that led to increased risks of breastfeeding termination were breastfeeding on demand in hospital and midwives teaching mothers how to initiate breastfeeding. Similarly, breastfeeding duration was shorter for women who did not experience all of the BFHI practices (19) (see Table 3).

Four retrospective studies comprised two chart reviews and two telephone surveys (4, 7, 14, 17).

Boldey and Powers (7) conducted chart reviews for 10 mothers who used nipple shields. The reasons for nipple shield use were the baby’s inability to grasp the areola (7/10), nipple soreness (1/10), and both of the aforementioned causes (2/10). The duration of shield use ranged from 2 weeks to 3.5 months, and all infants eventually quit nursing through the shield. All babies had appropriate weight gain at the 3–8 day, 3 week, 2 month, and 4 month weight check. Nine mothers were extremely positive about using the nipple shield to help in their situations, while one woman felt the shield was inconvenient, but she admitted that the tool helped her breastfeed (7) (see Table 3).

Wilson-Clay (4) also performed chart reviews for 32 women who received nipple shields from a lactation clinic. The most common problems at consultation were breast refusal (69%), difficulty with latch (25%), and sore nipples (6%). The duration of shield use varied among the study population. A total of 38% of mothers (12/32) weaned nipple shield use during the initial crisis period (<6 weeks), 56% (18/32) weaned after 6 weeks, and 6% (2/32) of women fed their infants human milk by bottle (4) (see Table 3).

Brigham (14) interviewed 51 clients of the Breastfeeding Center at Evergreen Hospital, who were given a nipple shield by telephone. The reasons for nipple shield use included difficulty with latch [37/51 (73%)], sore nipples [5/51 (10%)], and both of the aforementioned causes [9/51 (18%)]. The average age of infants when the nipple shield was first used was 6.1 days, ranging from 1 to 42 days, and the average length of shield use was 26.7 days, ranging from 2 days to 4.5 months. The majority of respondents [44/51 (86%)] reported that the nipple shield helped them continue to breastfeed. None of the women surveyed identified insufficient milk supply or poor infant growth patterns with nipple shield use (14) (see Table 3).

Powers and Tapia (17) conducted a telephone survey that assessed mothers’ perceptions regarding use of a nipple shield and its impact on their breastfeeding experience. Two hundred two women who had discontinued nipple shield use for at least 1 week at the time of the survey were included in this study. The reasons for nipple shield use included short or flat nipples [125/202 (62%)], infant’s disorganized suck [88/202 (43%)], and sore nipples [49/202 (23%)], with 46% of mothers giving more than one reason for using a shield. Nipple shield use began between the 1st and 42nd day of the infant’s life, and the median duration of use was 2 weeks. A total of 11% (22/202) of mothers reported that the infant could have nursed without the nipple shield at any time, but they chose to use the shield for nipple pain or general comfort. Almost all of the women surveyed [178/202 (88%)] felt that the nipple shield helped them succeed at breastfeeding (17) (see Table 3).

### Health Professionals’ Experiences

One study reported the health professionals’ experiences with nipple shield use (20). Eglash et al. (20) created a web-based survey to collect detailed descriptive data from respondents who work with breastfeeding mothers in diverse settings. The study population consisted of 490 physicians and other health professionals specialized in breastfeeding management, 92% (451/490) of whom had used nipple shields in their practices before. Their most common reasons for recommending nipple shield use were to help <35 weeks premature infants latch and nurse, to accommodate flat or inverted nipples, and to act as a method to transition an infant from bottles to the breast. The most common concerns among participants about nipple shield use were the “lack of follow-up by those introducing the nipple shield,” “inappropriate reasons for using nipple shields,” and “maternal inconvenience of using nipple shields.” Respondents reported that they hear mixed responses from women who have used nipple shields, such as “they are helpful,” “the nipple shield is convenient,” “the nipple shield is inconvenient,” and “(they) cannot wait to get rid of the nipple shield” (20) (see Table 4).

**Table 4:** **Health professionals’ experiences with nipple shield usage**.

Author	Study population	Methods	Outcomes
Eglash et al. (20)	• 490 physicians and other health professionals specialized in breastfeeding management • Most respondents were board certified in lactation [412/490 (79%)] • Most prevalent occupations – Lactation consultants [270/490 (52%)] – Nurses [125/490 (24%)] – Physicians [43/490 (8%)] – La Leche League Leader [29/490 (6%)] • 92% (451/490) of participants have used nipple shields in their practices	• A web-based anonymous survey was advertised via internet – Remained available online for a period of 3 weeks • Collected detailed descriptive data from respondents who work with breastfeeding mothers in diverse settings • Data from the survey were based on subjective recall of the health prof essionals’ experiences with nipple shields • Subjects were asked about – Their most common reasons for recommending nipple shield use – Their most common concerns about nipple shield use – What they typically hear from breastfeeding women who have used nipple shields	• Reasons for nipple shield use – To help <35 weeks premature infants latch and nurse – Flat/inverted nipples (16%) – Method to transition an infant from bottles to breast (14%) • Concerns for nipple shield use – “Lack of follow-up by those introducing the nipple shield” – “Inappropriate reasons for using nipple shields” – “Maternal inconvenience of using nipple shields” • Maternal responses for nipple shield use – “They are helpful” – “The nipple shield is convenient” – “The nipple shield is inconvenient” – “Cannot wait to get rid of the nipple shield”

## Discussion

There are many benefits to nipple shields. The use of a nipple shield can maintain breastfeeding, along with providing the mother a sense of accomplishment (2, 14). This ensures that the infant is comfortable and oriented to the breast (14). Additionally, nipple shields can help establish a breastfeeding relationship, contributing to overall mother–infant health (2). Brigham has found that nipple shields tend to be the least costly solution both financially and emotionally to families. As well, the shield is not seen when breastfeeding, enabling mothers and their babies to resemble any other nursing team. This appearance can be crucial to new parents who need a simple and discreet feeding plan (14).

Moreover, nipple shields can compensate for immature feeding behaviors, such as short, ineffective sucking bursts and falling asleep immediately after being positioned at the breast in premature infants (16, 21). The design of the nipple shield seems to compensate for weak intraoral suction pressures (16). Since the shield creates a nipple shape in the baby’s mouth, it enables the infant to draw milk through expression with minimal suction, improving milk ejection and transfer. The firm artificial nipple structure is maintained even during pauses in sucking bursts, ensuring that the baby stays attached to the nipple and does not slip off. Furthermore, once the shield is correctly positioned over the nipple and the infant begins to suck, negative pressure seems to be produced in the chamber between the maternal nipple’s tip and the shield’s interior. These pressures may balance out the infant’s weak suck and allow the milk to be accumulated in the shield during pauses in sucking, which will then be available to the baby immediately when sucking continues (16). Resultantly, shield use increases both the duration of sucking bursts and the volume of milk consumed during breastfeeding for babies born prematurely (22). In addition, after experiencing a difficult pregnancy which ended in a preterm birth and consequent hospitalization and separation of the baby from the family, many mothers of premature infants want to breastfeed (2). By helping these mothers breastfeed their infants, perhaps with the help of a nipple shield, they receive one expected and planned outcome of their pregnancy (2).

However, there are widespread negative attitudes toward nipple shields. In a breastfeeding guide for healthcare professionals, it states that “Many lactation experts consider the use of a [nipple] shield a sign of failure of proper lactation guidance” (23). There are three main reasons for discouraging nipple shield use: (1) nipple shields are thought to reduce milk transfer from the mother to the infant and prevent complete breast emptying; (2) they are considered addictive, such that infants may prefer the nipple shield rather than the breast, making it difficult to terminate its use; (3) incomplete breast emptying and an infant’s addiction to nipple shields is perceived to decrease the mother’s milk yield over time, causing early unplanned weaning (16). Other philosophical and scientific concerns include its similarities with bottles by acting as an artificial barrier between the infant and the breast (16); its support for an industry that makes breastfeeding “unnatural” (16); poor growth patterns in infants (24); prevention of the proper extension of the nipple back into the baby’s mouth, which might interfere with learning to suckle correctly (25); improper introduction of a shield to feed a baby before hospital discharge (11, 24); cause or worsening of sore nipples (11, 24); possible nipple trauma from pinching of the nipple and areola, especially with misuse (26); as well as reduced stimulation of the areola, which may interfere with prolactin and oxycotin release (24). Evidently, nipple shields remain a controversial issue in both the literature and clinical settings.

The reasons for varying durations of nipple shield use are not clear. It seems that a woman’s perception of her baby’s ease or difficulty with breastfeeding plays a role in the length of use of nipple shields. Some mothers have low tolerance for witnessing her infant struggle at the breast. Equivalently, women have different pain tolerances and abilities to cope with stress, which impacts how they deal with nipple soreness (7). Powers and Tapia discovered that a woman’s frustration with nipple shield use tends to be exceeded by the frustration of an infant that is unable to latch or that causes severe nipple pain (17).

Healthcare professionals should include evaluation of mothers’ nipples for elasticity during pregnancy and in the postpartum period to screen for and identify women at risk for lactation difficulty (4). In order to prevent potential inappropriate nipple shield use, clinical staff (e.g., nurses, neonatologists, pediatricians, lactation consultants) and parents need to be educated about the benefits and risks of nipple shields, newborn recovery, early breastfeeding, and elimination patterns versus genuine breastfeeding problems. Parents should be provided with early follow-up and resource phone numbers for breastfeeding assistance, which is especially important when in-hospital care is short (14). Likewise, public health should implement improved post-discharge support services and/or interventions that improve opinions toward breastfeeding in socio-cultural and economic groups to promote and encourage longer durations of breastfeeding (19). Care providers should also include the mother in the decision-making process, allowing her to make the choice that is the most beneficial for her and her infant (17).

The goal of lactation management is to offer individualized care and solutions leading to continued breastfeeding (14). For each problem, several paths may result in successful breastfeeding. Follow-up is the key to any lactation strategy. When lactation tools or techniques are initiated, including nipple shields, follow-up is necessary to assess the plan’s effectiveness, progress toward resolving the problem, and mother–infant satisfaction and comfort (14).

The findings from this review are very important in the field of lactation in many ways. Through examining the use of nipple shields, further insight is provided on the advantages and disadvantages of this practice, thus allowing clinicians and researchers to address improvements on areas that will benefit mothers and infants the most.

## Conflict of Interest Statement

The authors declare that the research was conducted in the absence of any commercial or financial relationships that could be construed as a potential conflict of interest.
